# "Poker" association of weekly alternating 5-fluorouracil, irinotecan, bevacizumab and oxaliplatin (FIr-B/FOx) in first line treatment of metastatic colorectal cancer: a phase II study

**DOI:** 10.1186/1471-2407-10-567

**Published:** 2010-10-19

**Authors:** Gemma Bruera, Alessandra Santomaggio, Katia Cannita, Paola Lanfiuti Baldi, Marianna Tudini, Federica De Galitiis, Maria Mancini, Paolo Marchetti, Adelmo Antonucci, Corrado Ficorella, Enrico Ricevuto

**Affiliations:** 1 Medical Oncology, S. Salvatore Hospital, University of L'Aquila, L'Aquila, Italy; 2 Medical Oncology, IDI, Rome, Italy; 3 Medical Oncology, S. Andrea Hospital, University La Sapienza, Rome, Italy; 4 General Surgery, S. Salvatore Hospital, L'Aquila, Italy

## Abstract

**Background:**

This phase II study investigated efficacy and safety of weekly alternating Bevacizumab (BEV)/Irinotecan (CPT-11) or Oxaliplatin (OHP) associated to weekly 5-Fluorouracil (5-FU) in first line treatment of metastatic colorectal carcinoma (MCRC).

**Methods:**

Simon two-step design: delta 20% (p_0 _50%, p_1 _70%), power 80%, α 5%, β 20%. Projected objective responses (ORR): I step, 8/15 patients (pts); II step 26/43 pts. Schedule: weekly 12 h-timed-flat-infusion/5-FU 900 mg/m^2^, days 1-2, 8-9, 15-16, 22-23; CPT-11 160 mg/m^2 ^plus BEV 5 mg/kg, days 1,15; OHP at three dose-levels, 60-70-80 mg/m^2^, days 8, 22; every 4 weeks.

**Results:**

Fifty consecutive, unselected pts < 75 years were enrolled: median age 63; young-elderly (yE) 24 (48%); liver metastases (LM) 33 pts, 66% Achieved OHP recommended dose, 80 mg/m^2^. ORR 82% intent-to-treat and 84% as-treated analysis. Median progression-free survival 12 months. Equivalent efficacy was obtained in yE pts. Liver metastasectomies were performed in 26% of all pts and in 39% of pts with LM. After a median follow-up of 21 months, median overall survival was 28 months. Cumulative G3-4 toxicities per patient: diarrhea 28%, mucositis 6%, neutropenia 10%, hypertension 2%. They were equivalent in yE pts. Limiting toxicity syndromes (LTS), consisting of the dose-limiting toxicity, associated or not to G2 or limiting toxicities: 44% overall, 46% in yE. Multiple versus single site LTS, respectively: overall, 24% versus 20%; yE pts, 37.5% versus 8%.

**Conclusion:**

Poker combination shows high activity and efficacy in first line treatment of MCRC. It increases liver metastasectomies rate and can be safely administered.

**Trial registration:**

Osservatorio Nazionale sulla Sperimentazione Clinica dei Medicinali (OsSC) Agenzia Italiana del Farmaco (AIFA) Numero EudraCT 2007-004946-34

## Background

Over the last 15 years, multiple active drugs were used in doublet or triplet combinations of chemotherapy and/or anti-targets, as first or subsequent lines of treatment [1-12] and increased overall survival of MCRC pts.

Doublet combinations of 5-FU or Capecitabine associated to CPT-11 or OHP or BEV achieve ORR 40%, progression free survival (PFS) 6-9 months and overall survival (OS) 15-22 months [1-11]. The addition of BEV to 5-FU or CPT-11/5-FU/Leucovorin (IFL) significantly increased, respectively: ORR up to 34.1% and 44.8%; PFS up to 8.8 and 10.6 months; median OS up to 17.9 and 20.3 months [8-11]. Advantages were also demonstrated with the addition of BEV to OHP-containing regimens; 5-FU (bolus plus infusion) or Capecitabine and OHP plus BEV showed PFS 10 months and OS 26 months [13-16]. Triplet association of chemotherapy, FOLFOXIRI, also demonstrated, in a phase III study, a statistically significant increase in activity and efficacy: ORR 60%, PFS 9.8 months, OS 22.6 months [17]. Another phase III study failed to demonstrate any increase [18]. As the activity of MCRC treatment progressively increased using triplet combinations, liver-metastasectomies also raised up to 8-11% of MCRC pts [17,18].

A major problem concerning the addition of more drugs in a chemotherapy combination is designing a proper schedule assuring the balance between dose intensity (DI) of each drug and tolerability. We previously showed that 12-hour (10^PM ^to 10^AM^) timed-flat-infusion (TFI) of 5-FU associated to CPT-11 can be safely administered at high 5-FU/DI without Leucovorin; ORR was 40%, median PFS 10 months, median OS 21 months [19]. The 12-hour (10^PM ^to 10^AM^) TFI/5-FU infusion traces the 12-hour circadian-timed infusion of 5-FU [3,20,21]. Recently, we designed the triplet schedule FIr/FOx by splitting TFI/5-FU weekly with alternating CPT-11 or OHP: ORR was 66.7%, median PFS was 12 months, median OS 20 months [22].

This present phase II study proposes FIr-B/FOx or "Poker" association (so called because it represents the combination of four drugs), adding Bevacizumab to this triplet chemotherapeutic schedule, in order to assess its activity, efficacy and safety.

## Methods

### Patient Eligibility

Pts were eligible if they had histologically confirmed diagnosis of measurable MCRC; age 18-75 years; World Health Organization (WHO) performance status ≤ 2; adequate hematological, renal and hepatic functions; life expectancy more than 3 months.

Ineligibility criteria: pregnancy and breast-feeding; uncontrolled severe diseases; cardiovascular disease (uncontrolled hypertension, uncontrolled arrhythmia, ischemic cardiac diseases in the last year); thromboembolic disease, coagulopathy, preexisting bleeding diatheses; proteinuria > 1 g/24 h urine; surgery within the previuos 28 days before; previous adjuvant chemotherapy or radiotherapy completed less than 6 months before.

The study was approved by the Local Ethical Committee (Comitato Etico, Azienda Sanitaria Locale n.4 L'Aquila, Regione Abruzzo, Italia) and conducted in accordance with the Declaration of Helsinki. All patients provided written, informed consent.

### Methods

#### Schedule

This was a single-arm, multicenter phase II study evaluating activity of weekly alternating 5-FU, CPT-11, BEV and OHP (FIr-B/FOx) as first-line treatment of MCRC.

FIr-B/FOx or "Poker" association consisted of 5-FU associated to alternating CPT-11/BEV or l-OHP according to the following weekly schedule: TFI/5-FU (Fluorouracil Teva^®^, Teva), 900 mg/m^2^/die, over 12-hour (from 10:00 p.m to 10:00 a.m.), days 1-2, 8-9, 15-16 and 22-23; CPT-11 (Campto^®^, Pfizer), 160 mg/m^2^, administered over 90 minutes as an intravenous infusion in 250 ml of NaCl 0.9%, days 1 and 15; BEV (Avastin^®^, Roche), 5 mg/kg, administered over 90 minutes at the first, 60 minutes at the second and 30 minutes from the third time, intravenous infusion in 100 ml of NaCl 0.9%, days 1 and 15; l-OHP (Eloxatin^®^, Sanofi-Aventis) over 2-hours as an intravenous infusion in 250 ml of dextrose 5%, at the dose of 60-70-80 mg/m^2^, days 8 and 22. Cycles repeated every 4 weeks. 5-FU was administered by a portable pump (CADD Plus, SEVIT) using a venous access device.

#### Study design

Physical examination and routine laboratory studies were performed at baseline and every week on-treatment, including complete blood cell count, electrolytes, liver and renal function tests, urine examination and coagulation function; tumor markers every cycle; electrocardiogram every two weeks and echocardiogram at baseline, and every 3 cycles.

Primary end-point was ORR; secondary end-points were PFS, toxicity, OS. ORR was evaluated according to RECIST criteria [23]; PFS and OS using Kaplan and Meier method [24]. PFS was defined as length of time between the beginning of treatment and disease progression or death (resulting from any cause) or to last contact; OS as length of time between the beginning of treatment and death or to last contact.

Clinical evaluation of response was planned by CT-scan; PET was added based on investigators' assessment; objective responses were confirmed three months later. Follow-up was scheduled every three months up to disease progression or death. Toxicity was registered every week according to National Cancer Institute Common Toxicity Criteria (NCI-CTC, version 3.0). DLT was defined as grade 3-4 non-haematological toxicity (mainly represented by diarrhea, mucositis, neurotoxicity, hand-foot syndrome, asthenia), grade 4 hematologic toxicity, febrile neutropenia, or any toxicity determining a > 2 weeks treatment delay.

The concept of limiting toxicity syndromes (LTS), consisting of at least a dose-limiting toxicity (DLT) associated or not to other limiting or G2 toxicities, was established. These were classified as: LTS in single site (LTS-ss), if characterized only by the DLT; LTS in multiple sites (LTS-ms), if characterized by ≥ 2 DLTs or a DLT associated to other, at least G2, non-limiting toxicities.

#### Statistical design

This phase II study was planned according to two-steps Simon's design [25]: assuming as minimal interesting activity an ORR 50%, 8 objective responses among the first 15 enrolled pts were necessary for the first-step; to verify the alternative hypothesis of ORR 70%, 26 objective responses among the total 43 pts enrolled were necessary; power (1 - β) 80%; error probability α 5%. p_0 _was considered as the activity of triplet combinations in MCRC (CPT-11/OHP/5-FU or BEV/CPT-11/5-FU) [17,18,20,11]; p_1 _as the projected ORR using the present Poker combination, increasing the activity ≥ 20%.

In the first step of the study, a dose-finding was concomitantly developed to verify recommended OHP dose by 3 escalating steps at 60, 70, 80 mg/m^2^, according to an intra-and inter-patient approach [26].

## Results

### Patient Demographics

From February 2006 to March 2009, 50 consecutive, unselected pts were enrolled (Table 1): Male/Female ratio, 31/19; median age, 63 years; 24 (48%) young-elderly pts (65 < 75 y); WHO Performance Status 0/1-2, 48/2; metastatic disease metachronous in 15 pts, synchronous in 35 pts. Metastatic sites: liver 33 pts (66%), lung 10 pts (20%), lymph nodes 17 pts, (34%); local recurrence 10 pts (20%). Metastatic site was single in 32 pts (64%), multiple in 18 pts (36%). Single metastatic sites were: liver 22 pts (44%), lung 3 pts (6%), lymph nodes 3 pts (6%), local recurrence 4 pts (8%). Liver metastases were single in 11 pts (22%) and multiple in 22 pts (44%).

**Table 1:** Patients' features

	Total N. (%)
No. of patients	50

Sex	
Male/Female	31/19

Age, years	
median	63
range	40-73
≥ 65 years	24 (48)

WHO Performance Status	
0	48 (96)
1-2	2 (4)

Metastatic disease	
metachronous	15 (30)
synchronous	35 (70)

Primary tumor	
colon	24 (48)
rectum	26 (52)

Sites of metastases	
liver	33 (66)
lung	10 (20)
lymph nodes	17 (34)
local	10 (20)
Other	5 (10)

No. of involved sites	
1	32 (64)
≥ 2	18 (36)

Single metastatic sites	
liver	22 (44)
lung	3 (6)
lymph nodes	3 (6)
local	4 (8)

Liver metastases	
single	11 (22)
multiple	22 (44)

Previous adjuvant chemotherapy:	9 (18)
FA/5-FU bolus	4 (8)
Capecitabine	1 (2)
FOLFOX4	4 (8)

Previous radiotherapy:	6 (12)
RT alone	2 (4)
RT+CT (5-FU c.i.)	3 (6)
RT+CT (XELOX)	1 (2)

Abbreviation: WHO, World Health Organization; c.i., continous infusion.

### Dose finding

In the first step of the study, a concomitant dose-finding was conducted in order to assess the recommended OHP dose. At the first dose-level, 9 pts were enrolled and 12 cycles were administered; a DLT, G3 diarrhea, was observed in 1 out of 9 pts (11%). At the second dose level, 11 pts were treated (3 new pts); no DLT was observed. At the third dose level, 14 pts were treated (3 new pts); a DLT (7%) was observed, characterized by G3 mucositis (G3 stomatitis and G2 diarrhea, associated with G2 hypoalbuminemia). Thus, 2 DLTs were observed out of 15 pts (13%) and out of 37 cycles of treatment (5%). The OHP dose-finding established that the maximum tolerated dose (MTD) was not reached at the third dose level [Additional file 1: Supplemental Table S1]; thus, the recommended dose of OHP was 80 mg/m^2 ^day 8 and 22, every 4 weeks.

### Activity and efficacy

The preliminary analysis of efficacy, according to the two-steps Simon's design, was conducted among the first 15 patients: 14 patients were evaluable as treated and one patient did not received at least three cycles of treatment. ORR was 93% (α 0.05, CI ± 13) (14 objective responses): 12 partial responses (80%); 2 complete responses (13%); 1 progressive disease (7%) [Additional file 1: Supplemental Table S2].

**Figure 1:**
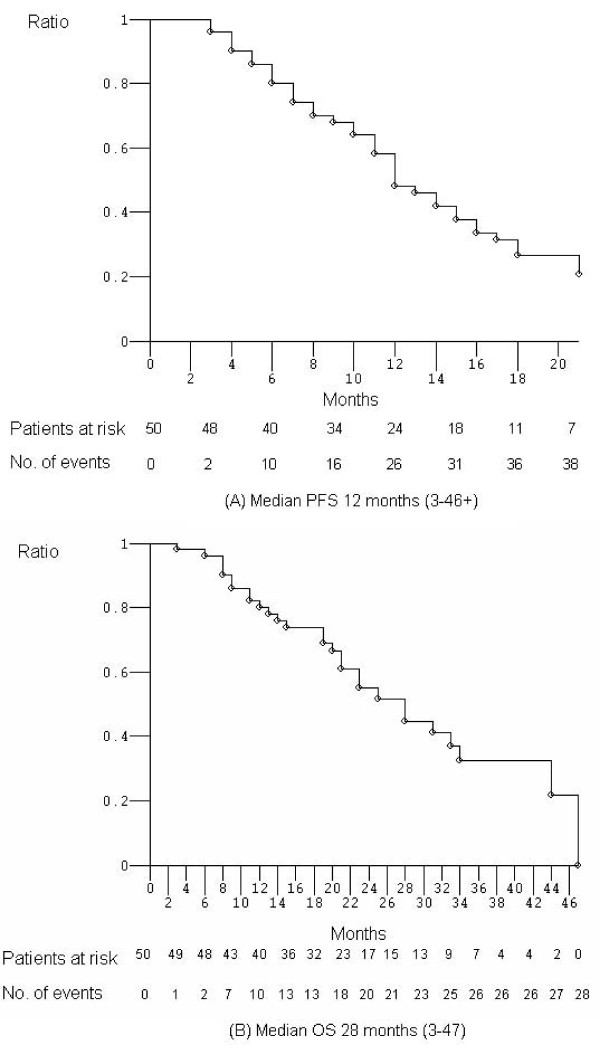
**Kaplan-Meier survival estimate. (A)** Progression-free survival **(B) **Overall survival. Abbreviation: PFS, progression-free survival; OS, overall survival.

Overall, 50 pts were enrolled to achieve 43 evaluable pts (Table 2). In the intent-to-treat analysis 49 pts were evaluable (one patient lost to follow-up): ORR was 82% (α 0.05, CI ± 11). We observed 40 objective responses: 36 partial responses (73%) and 4 complete responses (CR 8%); 2 stable diseases (4%); 7 progressive diseases (14%). Disease control rate was 86% (α 0.05, CI ± 10). In the as-treated analysis, 43 pts were evaluable: 6 pts did not receive at least three cycles of treatment, due to limiting gastrointestinal toxicity (3 patients) requiring treatment discontinuation in 2 of these, surgical resection of liver metastasis (1 patient) and of local recurrence (1 patient), clinical evidence of progressive disease (1 patient). ORR was 84% (α 0.05, CI ± 11). We observed 36 objective responses: 32 partial responses (75%) and 4 CR (9%); 2 stable diseases (5%); 5 progressive diseases (12%). Disease control rate was 88% (α 0.05, CI ± 10). After a median follow-up of 21 months (Figure 1), median PFS was 12 months (3-46+): 38 events occurred and 12 pts (24%) were progression-free > 12 months. Median OS was 28 months (3-47): 28 events occurred and 22 pts (44%) were alive; 80% of pts (40 pts) were alive > 12 months.

**Table 2:** Activity and efficacy data

	Intent-to-treat Analysis		As-treated Analysis	
	**No**	**%**	**No**	**%**

**Enrolled patients**	50	100	50	100

**Evaluable patients**	49	98	43	86

**Objective Response**	40	82 (CI ± 11)	36	84 (CI ± 11)
Partial Response	36	73	32	75
Complete Response	4	8	4	9

**Stable Disease**	2	4	2	5

**Progressive Disease**	7	14	5	12

**Median Progression-free survival, months**	12			
Range	3-46+			
Progression events	38	76		
		
**Median Overall Survival, months**	28			
Range	3-47			
Deaths	28	56		
		
**Liver metastasectomies**	13			
No/Overall patients (50)		26		
No/Patients with liver metastases (33)		39		
No/Patients with liver-only metastases (22)		54		

Among the 24 young-elderly pts, in the intent-to-treat analysis, ORR was 79% (α 0.05, CI ± 12): 16 partial responses (67%) and 3 CR (12.5%); 5 progressive disease (21%). In the as-treated analysis, 20 young-elderly pts were evaluable; ORR was 80% (α 0.05, CI ± 12). After a median follow-up of 18.5 months, median PFS was 11 months (3-46+): 20 events occurred and 4 pts were progression-free. Median OS was 20 months (6-46+): 16 events occurred and 8 pts were alive.

Liver metastasectomies were performed in 13 pts of the 50 enrolled pts (26%); 13 of the 33 pts with liver metastases (39%) (Table 2). Twelve metastasectomies out of 22 pts with liver-only metastases (54%) were performed: 6 in 9 pts with single liver metastasis (66%); 6 in 13 pts with multiple liver metastases (46%). In one patient with single liver metastasis associated with lung metastases, double metastatic resections were performed. Overall, R0 liver resections were 11 (84.6%), R1 resections were 2 (15.4%). Among the 22 pts with liver-only metastases, 5 pts had initially resectable disease (23%), 2 metastasectomies were performed and 2 pts showed a cCR; 6 pts had initially unresectable disease (27%), 3 metastasectomies were performed and 1 patient showed a cCR; 11 pts (50%) had potentially resectable liver metastases and 7 metastasectomies were performed. No surgery-related complications were reported. Hepatic steatosis was observed in 3 pts (25%). Liver metastases showed necrotic areas between 10 and 100% in 6 pts (50%). In 2 pts with multiple liver-only metastases a pathologic CR was obtained (6% of pts with liver metastases and 15% of liver metastasectomies).

Overall, 4 clinical and 2 pathologic CRs (12.5%) were reported; 1 patient showed a progressive disease at 17 months; 5 pts were progression-free at 46, 37, 19, 17 and 12 months, respectively.

Twenty-six pts (52%) received, at least, a second line treatment: FIr-B/FOx association in 4 pts (8%), who showed a long-lasting PFS; Cetuximab-containing treatment in 15 pts (30%): Bev-containing in 2 pts (4%); triplet-chemotherapy (FIr-FOx) in 1 patient (2%); Panitumumab in 1 patient (2%); Capecitabine alone in 3 pts (6%).

### Dose-intensity

Median number of administered cycles was 5 (range 2-9).

Median received dose intensities (rDI) per cycle were: 5-FU 1487 (480-1800) mg/m^2^/w, 82.6% of projected-DI (pDI); CPT-11 66.7 (25-80) mg/m^2^/w, 83% of pDI; l-OHP 32 (8-40) mg/m^2^/w, 80% of pDI; BEV 2.1 (1-2.5) mg/kg/w, 84% of pDI (Table 3). Median rDI per patient were: 5-FU 1500 (955-1800) mg/m^2^/w, 83% of pDI; CPT-11 66 (45-80) mg/m^2^/w, 82.5% of pDI; l-OHP 32.5 (20.5-40) mg/m^2^/w, 81% of pDI; BEV 2 (1.5-2.5) mg/kg/w, 80% of pDI. In young elderly pts, median rDIs per cycle were: 5-FU 1420 (480-1800) mg/m^2^/w, 80% of pDI; CPT-11 61 (25-80) mg/m^2^/w, 76% of pDI; l-OHP 31.5 (8-40) mg/m^2^/w, 79% of pDI; BEV 2 (1-2.5) mg/kg/w, 80% of pDI.

**Table 3:** Dose-intensity

		All patients		Young-elderly patients	
		**DI/cycle mg/m**^**2**^**(or Kg)/w**		**DI/cycle mg/m**^**2**^**(or Kg)/w**	

	**Projected DI mg/m**^**2**^**(o Kg)/w**	**Median (Range)**	**Received DI (%)**	**Median (Range)**	**Received DI (%)**

**5-FU**	1800	1487	82,6	1420	80
		(480-1800)		(480-1800)	

**CPT-11**	80	66.7	83	61	76
		(25-80)		(25-80)	

**l-OHP**	40	32	80	31.5	79
		(8-40)		(8-40)	

**BEV**	2.5	2.1	84	2	80
		(1-2.5)		(1-2.5)	

Abbreviation: DI, dose-intensity; 5-FU, 5-Fluorouracil; CPT-11, Irinotecan; l-OHP, Oxaliplatin; BEV, Bevacizumab.

### Toxicity

Table 4 describes cumulative toxicities in enrolled pts and in 247 administered cycles. Two out of 50 patients (4%) discontinued FIr-B/FOx treatment due to limiting toxicity (grade 3 diarrhea). Cumulative G3-4 toxicities, by pts, were: nausea 3 pts (6%), vomiting 2 pts (4%), diarrhea 14 pts (28%), stomatitis/mucositis 3 pts (6%), erythema 1 patient (2%), asthenia 3 pts (6%), hypertension 1 patient (2%), hypokalemia 1 patient (2%), hypertransaminasemy 2 pts (4%), alopecia 3 pts (6%), neutropenia 5 pts (10%). The prevalent DLT was diarrhea, equally distributed among young-elderly and non-elderly pts: 6 pts (25%) and 8 pts (31%), respectively. Other G3-4 toxicities were mostly observed in the 24 young elderly pts, particularly nausea 2 pts, vomiting 2 pts, stomatitis/mucositis 3 pts, erythema 1 patient, asthenia 3 pts, alopecia 3 pts and neutropenia 3 pts. Cumulative G2 toxicities, by pts, were: nausea 15 pts (30%), vomiting 6 pts (12%), diarrhea 12 pts (24%), hypoalbuminemia 1 patient (2%), stomatitis/mucositis 2 pts (4%), asthenia 20 pts (40%), neurotoxicity 5 pts (10%), hypertension 4 pts (8%), hematuria 1 patient (2%), epistaxis 2 pts (4%), hypertransaminasemy 2 pts (4%), alopecia 9 pts (18%), neutropenia 14 pts (28%), thrombocytopenia 1 patient (2%). No case of thrombosis, hemorrhage/bleeding, cardiac or cerebrovascular ischemia, G4 neutropenia, febrile neutropenia, severe thrombocytopenia, or toxic deaths were observed.

**Table 4:** Cumulative toxicity

	Patients				Cycles			
***Number***	**50**				**247**			

**NCI-CTC Grade**	**1**	**2**	**3**	**4**	**1**	**2**	**3**	**4**

Nausea (%)	23 (46)	15 (30)	3 (6)	-	81 (33)	23 (9)	4 (2)	-

Vomiting (%)	10 (20)	6 (12)	2 (4)	-	19 (8)	9 (4)	2 (1)	-

Diarrhea (%)	20 (40)	12(24)	14 (28)	-	76 (30)	28 (11)	15 (6)	-

Hypoalbuminemia (%)	2 (4)	1 (2)	-	-	2 (1)	1 (0.5)	-	-

Constipation (%)	17 (34)	1 (2)	-	-	22 (9)	1 (0.5)	-	-

Stomatitis/mucositis (%)	16 (32)	2 (4)	3 (6)	-	29 (12)	3 (1)	3 (1)	-

Erythema (%)	1 (2)	-	1 (2)	-	3 (1)	-	1 (0.5)	-

Asthenia (%)	13 (26)	20 (40)	3 (6)	-	48 (19)	38 (15)	3 (1)	-

Neurotoxicity (%)	36(72)	5 (10)	-	-	126(51)	6 (2)	-	-

Hypertension (%)	15 (30)	4 (8)	1 (2)	-	27 (11)	4 (2)	1 (0.5)	-

Hypotension (%)	1 (2)	-	-	-	1 (0.5)	-	-	-

Hematuria (%)	2 (4)	1 (2)	-	-	3 (1)	1 (0.5)	-	-

Gengival recession/gengivitis (%)	7 (14)	-	-	-	10 (4)	-	-	-

Rhinitis (%)	38 (76)	-	-	-	110(44.5)	-	-	-

Epistaxis (%)	31 (62)	2 (4)	-	-	68 (27.5)	2 (1)	-	-

HFS (%)	2 (4)	-	-	-	2 (1)	-	-	-

Headache (%)	6 (12)	-	-	-	9 (4)	-	-	-

Hypokalemia (%)	3 (6)	-	1 (2)	-	3 (1)	-	1 (0.5)	-

Hypertransaminasemy (%)	3 (6)	2 (4)	1 (2)	1 (2)	9 (4)	6 (2)	1 (0.5)	1 (0.5)

Hyperpigmentation (%)	6 (12)	2 (4)	-	-	14 (6)	5 (2)	-	-

Fever without infection (%)	10 (20)	-	-	-	10 (4)	-	-	-

Alopecia (%)	5 (10)	9 (18)	3 (6)	-	11 (4)	17 (7)	7 (3)	-

Anemia (%)	7 (14)	4 (8)	-	-	16 (6)	4 (2)	-	-

Leucopenia (%)	13 (26)	17 (34)	-	-	49 (20)	26 (10.5)	-	-

Neutropenia (%)	9 (18)	14 (28)	5 (10)	-	35 (14)	32 (13)	8 (3)	-

Thrombocytopenia (%)	7 (14)	1 (2)	-	-	16 (6)	1 (0.5)	-	-

Abbreviation: NCI-CTC, National Cancer Institute Common Toxicity Criteria.

Overall, LTS were observed in 22 pts (44%) (Table 5); 11 out of 24 young-eldely pts (46%). LTS-ss in 10 pts (20%); LTS-ms in 12 pts (24%). LTS-ms characterized by DLT associated to other, at least G2, non-limiting toxicities were detected in 10 pts (20%); ≥ 2 DLTs in 2 pts (4%). Among the 24 young-elderly pts the distribution of LTS was: LTS-ss, 2 pts (8%), LTS-ms, 9 pts (37.5%). LTS-ms were characterized by: DLT associated to other, at least G2, non-limiting toxicities, 7 pts (29%); ≥ 2 DLTs, 2 pts (8%). The 10 LTS-ss were characterized by [Additional file 1: Supplemental Table S3]: G3 diarrhea, 5 pts; G3 asthenia, 1 patient; G3 hypertension, 1 patient; G3 hypertransaminasemy, 1 patient; G3 neutropenia, 1 patient; G1 thrombocytopeny for > 2 weeks, 1 patient. The 10 LTS-ms, characterized by DLT associated to other, at least G2, non-limiting toxicities, were characterized by: G3 diarrhea associated with G2-3 nausea and/or G2-3 vomiting, 4 pts; G3 diarrhea associated with G2 vomiting and G2 neurotoxicity, 1 patient; G3 diarrhea associated with G2 stomatitis/mucositis and G2 asthenia, 1 patient; G3 diarrhea associated with G3 nausea and G2 asthenia, 1 patient; G3 diarrhea associated with G2 epistaxis, 1 patient; G4 hpertransaminasemy associated with G2 diarrhea, G2 nausea and G2 anemia, 1 patient; G3 stomatitis/mucositis and G2 asthenia, 1 patient. The 2 LTS-ms, with double DLTs, were observed in young-elderly pts and characterized by: limiting diarrhea associated with mucositis and mucositis associated with erythema.

**Table 5:** Limiting Toxicity Syndromes (LTS): overall and in young-elderly patients

	Overall		Young-elderly	
	**N**.	**%**	**N**.	**%**

**Patients**	50	100	24	100

**Limiting Toxicity Syndromes (LTS)**	22	44	11	46

**LTS single-site (LTS-ss)**	10	20	2	8

**LTS multiple-sites (LTS-ms)**	12	24	9	37.5

Single DLT plus G2-3	10	20	7	29

Double DLTs	2	4	2	8

Abbreviation: DLT, dose-limiting toxicity; G, grade.

## Discussion

The present phase II study proposing FIr-B/FOx association in first line treatment of consecutive, unselected MCRC pts, reached the primary endpoint: ORR 82% in the ITT and 84% in the as-treated analysis. After a median follow-up of 21 months, median PFS was 12 months and 24% of pts were progression-free > 12 months; median OS was 28 months and 80% of pts (40 pts) were alive > 12 months.

The subsequent generations of randomized studies [8,10,11,13,14,17,18] showed increased activity and efficacy starting from ORR ≤ 20%, PFS 5 months and OS ≤ 14 months of 5-FU alone. Doublets consisting of CPT-11, or OHP, or BEV associated to 5-FU or Capecitabine gained ORR 20.0-47.0%, PFS 5.9-9.0 months and OS 15.1-21.5 months, without demonstrating differences between 5-FU/BEV associations compared to 5-FU or Capecitabine with CPT-11 or OHP. Triplets consisting of chemotherapeutic drugs or doublets plus BEV obtained ORR 39.0-66.0%, PFS 8.3-10.6 months and OS 20.3-26.1 months. In particular, the addition of a third drug, either BEV or OHP, equivalently increased the efficacy of doublet combination associating 5FU/CPT-11; 5-FU or Capecitabine and OHP plus BEV shows OS 20.4-26.1 months [13,14]. Based on these data, a phase II study of four drug association, adding BEV to FOLFOXIRI, was recently proposed [27].

We previously showed that doublet and triplet chemotherapy using TFI/5-FU, without Leucovorin, according to the present schedule [19,20], obtained equivalent efficacy to other reported schedules (ORR 40% and 66.7%, PFS 10 and 12 months, OS 21 and 20 months, respectively) and demonstrated a good tolerability profile [19,20,28]. In this scenario, Poker combination, adding BEV to FIr-FOx association, in first line treatment of MCRC, increased activity compared to triplet associations and it also increased efficacy; the 48% young elderly pts enrolled showed equivalent activity and efficacy.

Randomized studies of doublets (5-FU plus CPT-11 or OHP) adding Cetuximab (an EGFR-inhibitor) in EGFR-overexpressing pts, showed equivalent efficacy to other triplet combinations [29,30]; in these studies, k-ras wild-type status was reported as a statistically relevant predictive biomarker of higher activity and efficacy. Preliminary data of a phase III trial may also confirm this with Panitumumab plus FOLFOX4 [31]. The effectiveness of BEV-containing treatments was maintained in k-ras wild-type as it was in k-ras mutated pts [32].

Liver metastasectomies were performed in 26% of MCRC pts and 39% of pts with liver metastases. Moreover, 54% of pts with liver-only metastases and 50% of pts with initially unresectable liver metastases underwent surgical resection. Liver metastasectomies were reported in 8-11% of pts treated with triplet chemotherapy (36% in liver-only pts) [17,18] and in 7.6% of pts in BEV-containing associations (15.2% in liver-only pts) [16]; in pts with potentially curable liver metastases, they were 92.8% [15]. In Cetuximab-containing associations [29,30], metastasectomies were performed in 7% and 4.7% of overall pts [29,30]; 9.8% of KRAS wt pts [30]. More, using neoadjuvant Cetuximab with either FOLFOX6 or FOLFIRI for unresectable colorectal liver metastases, metastasectomies were performed in 38% and 30% of pts, respectively [33]. Our data show that more active first line treatment of MCRC [34], such as Poker combination, contribute to increase efficacy also by raising surgical resection of liver metastases.

Median rDIs per cycle and per patient were > 80% for each drug; in young elderly pts, only the rDI of CPT-11 was lower than 80% (76%; 61 mg/m^2^/w). Cumulative G3-4 toxicities were prevalently represented by diarrhea (28%), stomatitis/mucositis (6%), asthenia (6%), hypertension (2%), hypertransaminasemy (4%), neutropenia (10%). Cumulative G3-4 toxicities reported with the schedule associating BEV to 5-FU/Leucovorin or IFL were represented by equivalent prevalence of, respectively: diarrhea 28.5% and 32.4%; hypertension 8.5% and 11%; thrombotic events 14.2% and 19.4% [8,11]. Cumulative G3-4 toxicities reported with FOLFOXIRI schedules [17,18] were prevalently represented by, respectively: diarrhea 20% and 27.7%, stomatitis/mucositis 5% and 5%, asthenia 6% and 5.6%, neutropenia 50% and 35%, febrile neutropenia 5% and 7%, neurotoxicity 2% and 5.8%. Thus, gastrointestinal toxicities were not quite different in patients treated with 5-FU and CPT-11 as doublet, or associated to BEV, or OHP, or BEV and OHP as in the present study. Preliminary safety data of the phase III trial evaluating BEV plus FOLFOXIRI versus BEV plus FOLFIRI, show cumulative G3-4 toxicities prevalently represented by diarrhea (18%), stomatitis/mucositis (8%), asthenia (4%), hypertension (2%), trombotic events (8%), neutropenia (53%), febrile neutropenia (6%); a treatment-related death was reported in 1 patient (gastrointestinal bleeding; 2%). Thus, FIr-B/FOx schedule determined only 10% G3-4 neutropenia, while FOLFOXIRI schedule, added or not to BEV [35,17], prevalently induced it (53% and 50%, respectively) and also febrile neutropenia (6% and 5%, respectively).

Cumulative G3-4 toxicities in young-elderly pts were prevalently represented by diarrhea (25%), hypertransaminasemy (4%), neutropenia 12.5%, stomatitis/mucositis (12.5%), asthenia (12.5%). Souglakos et al reported a significantly higher incidence of G3-4 diarrhea in elderly versus non-elderly pts either in the FOLFIRI or in the FOLFOXIRI arm [18]. In our study, DLT was observed in 44% of pts and in 46% of young-elderly pts, with no differences of cumulative G3-4 toxicities by pts and by cycles. The innovative clinical evaluation of LTS, consisting of at least the DLT associated or not to other G2 or limiting toxicities, was introduced to better evaluate, in the individual patient, the presence of DLT alone, LTS-ss, or the association of major toxicities in different sites, LTS-ms: overall, they were 20% and 24% respectively; among young-elderly pts, they were 8% and 37.5%, respectively. Most LTS-ms (2 double DLTs and 7 out of 10 DLT associated to other, at least G2, non-limiting toxicities) were observed in this subgroup. LTS-ms were mostly represented by diarrhea and/or stomatitis/mucositis associated to nausea, vomiting and/or asthenia.

## Conclusions

Randomized trials will confirm if intensive chemotherapy approaches, such as Poker association, increase efficacy, also by raising surgical resection of liver metastases, as first line treatment of MCRC. The present schedule is feasible, also in young-elderly pts, with manageable toxicity. Young-elderly pts show equivalent efficacy and cumulative toxicity, with a prevalence of LTS-ms associating the DLT with other moderate/severe toxicities.

## Competing interests

The authors declare that they have no competing interests.

## Authors' contributions

Study conception: ER. Study design: ER. Data acquisition: All authors. Data analysis and interpretation: GB, AS, KC, ER. Manuscript preparation: GB, AS. Final approval: All authors. Manuscript revising: GB, ER.

## Pre-publication history

The pre-publication history for this paper can be accessed here:

http://www.biomedcentral.com/1471-2407/10/567/prepub

## Supplementary Material

Additional file 1 **Oxaliplatin dose-finding, activity in the first step of the study (Simon's design) and Limiting Toxicity Syndromes (LTS)**. Supplemental Table S1 reports results of the dose-finding planned in order to assess the recommended Oxaliplatin dose. Supplemental Table S2 describes preliminary data of activity, in the first step of the study (Simon's two-step design). Supplemental Table S3 describes toxicities characterizing Limiting Toxicity Syndromes (LTS) in individual patients. Click here for file
