# Supplementary material for: "Poker" association of weekly alternating 5-fluorouracil, irinotecan, bevacizumab and oxaliplatin (FIr-B/FOx) in first line treatment of metastatic colorectal cancer: a phase II study
Source: BMC Cancer. 2010 Oct 19;10:567. doi: 10.1186/1471-2407-10-567 (PMC2972284; doi:10.1186/1471-2407-10-567)
Supplement: Additional file 1 — Oxaliplatin dose-finding, activity in the first step of the study (Simon's design) and Limiting Toxicity Syndromes (LTS). Supplemental Table S1 reports results of the dose-finding planned in order to assess the recommended Oxaliplatin dose. Supplemental Table S2 describes preliminary data of activity, in the first step of the study (Simon's two-step design). Supplemental Table S3 describes toxicities characterizing Limiting Toxicity Syndromes (LTS) in individual patients. [file 1471-2407-10-567-S1.PDF]

Additional file 1

Supplemental Table S1: Oxaliplatin dose-finding

Dose levels	CPT11 (mg/m^2 d1,15)-BEV (mg/kg d1,15)-l-OHP (mg/m^2 d8,22)-5-FU (mg/m^2/d d1-2, 8-9, 15-16, 22-23)	No. patients[a] (new patients)	No. cycles	No. Patients with DLT/ total patients (%)	No. New patients with DLT/new patients (%)	No. cycles with DLT/total cycles (%)	DLTs
I	900-160-5-60	9 (9)	12	1/9 (11%)	1/9 (11%)	1/12 (8%)	G3 Diarrhea
II	900-160-5-70	11 (3)	11	0/11 (-)	0/3 (-)	0/11 (-)	-
III	900-160-5-80	14 (3)	14	1/14 (7%)	0/8 (-)	5/14 (7%)	G3 Mucositis G3 Diarrhea

[a] intra- and inter- patient dose escalation;
Abbreviation: 5-FU, 5-Fluorouracil; CPT-11, Irinotecan; l-OHP, Oxaliplatin; BEV, Bevacizumab; DLT, dose-limiting toxicity.

Supplemental Table S2: Activity in the first step of the study (Simon's design)

	Intent-to-treat Analysis		As-treated Analysis	
	**No**	**%**	**No**	**%**
**Enrolled patients**	15	100	15	100
**Evaluable patients**	15	100	14	93
**Objective Response**	14	93	13	93
**Partial Response**	12	80	11	78.5
**Complete Response**	2	13	2	14
**Stable Disease**	-	-	-	-
**Progressive Disease**	1	7	1	7

Supplemental Table S3: Limiting Toxicity Syndromes (LTS)

Patients #	Age (years)	DLT	Associated Toxicity	
			DLT	G2-G3
**1**	51	Diarrhea G3	-	-
**2**	62	Diarrhea G3	-	-
**3**	71	Diarrhea G3	-	-
**4**	62	Diarrhea G3	-	-
**5**	58	Diarrhea G3	-	-
**6**	70	Asthenia G3	-	-
**7**	62	Hypertension G3	-	-
**8**	51	Hypertransaminasemy G3	-	-
**9**	55	Thrombocytopenia G1 for > 2 weeks	-	-
**10**	63	Neutropenia G3	-	-
**11**	68	Diarrhea G3	-	Vomiting G3
**12**	59	Diarrhea G3	-	Vomiting G2<br>Neurotoxicity G2
**13**	67	Diarrhea G3	-	Nausea G3<br>Asthenia G2
**14**	57	Diarrhea G3	-	Nausea G3
**15**	67	Diarrhea G3	-	Vomiting G2
**16**	65	Diarrhea G3	-	Epistaxis G2
**17**	40	Diarrhea G3	-	Nausea G2
**18**	66	Diarrhea G3	-	Stomatitis/mucositis G2<br>Asthenia G2
**19**	67	Stomatitis/mucositis G3	-	Asthenia G2
**20**	66	Hypertransaminasemy G4	-	Diarrhea G2<br>Nausea G2<br>Anemia G2
**21**	71	Diarrhea G3	Stomatitis/mucositis G3	Hypoalbuminemia G2
**22**	66	Stomatitis/mucositis G3	Erythema G3	-

Abbreviation: DLT, dose-limiting toxicity; G, grade.
